# Australian parents’ attitudes, perceptions and supply of alcohol to adolescents: a national cross-sectional survey

**DOI:** 10.1093/heapro/daae173

**Published:** 2024-12-09

**Authors:** Jacqueline A Bowden, Ashlea Bartram, Nathan J Harrison, Christina A Norris, Susan Kim, Simone Pettigrew, Ian Olver, Rebecca Jenkinson, Marina Bowshall, Caroline Miller, Robin Room

**Affiliations:** National Centre for Education and Training on Addiction (NCETA), Flinders University, Kaurna Country, GPO Box 2100, Adelaide, South Australia 5001, Australia; College of Medicine and Public Health, Flinders University, Flinders Health and Medical Research Institute, Kaurna Country, GPO Box 2100, Adelaide, South Australia 5001, Australia; National Centre for Education and Training on Addiction (NCETA), Flinders University, Kaurna Country, GPO Box 2100, Adelaide, South Australia 5001, Australia; College of Medicine and Public Health, Flinders University, Flinders Health and Medical Research Institute, Kaurna Country, GPO Box 2100, Adelaide, South Australia 5001, Australia; National Centre for Education and Training on Addiction (NCETA), Flinders University, Kaurna Country, GPO Box 2100, Adelaide, South Australia 5001, Australia; College of Medicine and Public Health, Flinders University, Flinders Health and Medical Research Institute, Kaurna Country, GPO Box 2100, Adelaide, South Australia 5001, Australia; National Centre for Education and Training on Addiction (NCETA), Flinders University, Kaurna Country, GPO Box 2100, Adelaide, South Australia 5001, Australia; College of Medicine and Public Health, Flinders University, Flinders Health and Medical Research Institute, Kaurna Country, GPO Box 2100, Adelaide, South Australia 5001, Australia; National Centre for Education and Training on Addiction (NCETA), Flinders University, Kaurna Country, GPO Box 2100, Adelaide, South Australia 5001, Australia; College of Medicine and Public Health, Flinders University, Flinders Health and Medical Research Institute, Kaurna Country, GPO Box 2100, Adelaide, South Australia 5001, Australia; The George Institute for Global Health, University of New South Wales, PO Box M201, Sydney, New South Wales 2050, Australia; School of Psychology, The University of Adelaide, Kaurna Country, North Terrace, Adelaide, South Australia 5000, Australia; Australian Institute of Family Studies, 40 City Road, Southbank, Victoria 3006, Australia; School of Public Health and Preventive Medicine, Monash University, 553 St Kilda Road, Melbourne, Victoria 3004, Australia; Burnet Institute, 85 Commercial Road, Melbourne, Victoria 3004, Australia; Preventive Health SA, Kaurna Country, 11/80 Grenfell Street, Adelaide, South Australia 5000, Australia; Health Policy Centre, South Australian Health and Medical Research Institute, Kaurna Country, North Terrace, Adelaide, South Australia 5000, Australia; School of Public Health, The University of Adelaide, Kaurna Country, North Terrace, Adelaide, South Australia 5000, Australia; Centre for Alcohol Policy Research, School of Psychology and Public Health, La Trobe University, Kingsbury Drive, Bundoora, Victoria 3086, Australia; Centre for Social Research on Alcohol and Drugs, Department of Public Health Sciences, Stockholm University, SE-106 91, Stockholm, Sweden

**Keywords:** parental supply, parents, adolescent health, alcohol, Australia

## Abstract

Parental supply of alcohol to adolescents is associated with increased risk of subsequent adolescent alcohol use and harms, so identifying factors associated with parents’ decision-making is critical. This study examined how parental supply is associated with attitudes toward adolescent alcohol use, perceived norms of parental supply, perceived behavioural control and perceived acceptable age to drink alcohol. A total of 1197 Australian parents with children aged 12–17 years completed an online cross-sectional survey assessing their parental supply behaviours, attitudes and perceptions in April 2022. Logistic regression was used to explore associations between attitudes, perceptions and parental supply of alcohol to their child. Forty-three percent of respondents nominated an acceptable age to drink a full drink of alcohol below 18 years, and 23% reported supplying a full drink of alcohol to their adolescent. Parents were more likely to report supplying a full drink of alcohol if they nominated an acceptable drinking age below 18 years (<16: adjusted odds ratio [AOR] = 14.75, 95% confidence interval [CI] = 8.23–26.42; 16–17: AOR = 5.68, 95% CI = 3.69–8.73), appraised alcohol as more beneficial (AOR = 1.31, 95% CI = 1.02–1.69) and less harmful (AOR = 0.49, 95% CI = 0.36–0.68) for adolescents, and perceived that parent friends (AOR = 2.91, 95% CI = 1.80–4.70) and other parents (AOR = 2.23, 95% CI = 1.37–3.62) supplied alcohol in unsupervised contexts. Perceived behavioural control was not associated with parental supply. These findings suggest there may be value in trialling interventions that target parents’ perceptions about the acceptable age to drink a full drink of alcohol, attitudes toward adolescent alcohol consumption, and perceived norms of parental supply to influence parents’ supply intentions.

**Contribution to Health Promotion**

- Parents are more likely to supply alcohol if they believe other parents supply alcohol.
- Parents who view alcohol as beneficial for adolescents are more likely to supply alcohol.
- Parents who view alcohol as harmful for adolescents are less likely to supply alcohol.
- Believing it is acceptable to drink alcohol under 18 years increases odds of supply.
- Targeting these factors in interventions may influence parents’ supply intentions

## INTRODUCTION

Many parents assume that providing alcohol to adolescents in supervised settings such as the home has a protective effect against future alcohol consumption (Jones, 2016). However, a growing body of longitudinal cohort studies (Mattick *et al*., 2017, 2018; Aiken *et al*., 2020) and systematic reviews (Ryan *et al*., 2010; Sharmin *et al*., 2017) provides compelling evidence that parental supply is associated with earlier alcohol initiation, risky drinking behaviour and higher levels of later alcohol use. Further, adolescents who are supplied alcohol by their parents in early adolescence are more likely than those not supplied alcohol to obtain alcohol via other sources in later years (Boland *et al*., 2020). Even parental provision of sips of alcohol increases the likelihood of binge drinking and alcohol-related harms, although the supply of increased alcohol quantities (i.e. full drinks) is associated with increased risk (Aiken *et al*., 2020).

A recent systematic review of the prevalence of parental supply to minors indicated that this practice is common across the globe, but with considerable variation found between studies, often reflecting measurement differences (e.g. prevalence ranged from 7% to 60% in samples with reporting from minors themselves and from 24% to 48% when reported by parents) (van der Kruk *et al*., 2023). Parental supply was reported to be most prevalent among minors who drink alcohol in New Zealand (60%), Australia (47%), Thailand (42%), and Sweden (42%; van der Kruk *et al*., 2023). In Australia, the setting of this study, parents are the most common source of alcohol to adolescents who drink (Scully *et al*., 2023). The proportion of Australian secondary students who have recently consumed alcohol reporting that they obtained their last alcoholic drink from parents has increased substantially from 29% in 1996 to 47% in 2022/2023 (White *et al*., 2000).

Reducing parental supply of alcohol is thus an important target for health promotion interventions. To inform effective interventions, it is important to understand what factors are associated with parents’ supply behaviours. This study examined parental supply of alcohol through the lens of a tailored Theory of Planned Behaviour (TPB) that incorporates the ‘social clock’: the concept that behaviours that may be considered deviant and concerning before a child reaches an ‘acceptable’ age, but appropriate or even expected after that age (Paglia and Room, 1998).

### Understanding parental supply through the TPB

The TPB posits that behavioural intentions predict behaviour, and that these intentions are shaped by individuals’ attitudes, norms and perceptions of behavioural control (Ajzen, 1991). The theory has been used to predict behaviours ranging from smoking cessation to recycling household waste, with a meta-analytic review of 185 studies finding that the model accounted for 27% of the variance in behaviour (Armitage and Conner, 2001). The theory has also been used to predict behaviours undertaken by parents to promote the health of their child, such as providing healthy meals (Hamilton *et al*., 2020).

With respect to parental supply of alcohol, the TPB has served as an explanatory framework to synthesize the literature (Jones, 2016). Jones found that attitudes toward adolescent alcohol use and perceived norms of parental supply were associated with intentions to supply alcohol (Jones, 2016). Another study found that parents’ perceived norms were inaccurate, with parents perceiving general community attitudes toward parental supply to be more liberal than their own (Jones and Francis, 2015). None of the studies identified by Jones directly addressed associations between parental supply and perceived behavioural control: the extent to which parents perceive that the provision of alcohol to adolescents is controllable (Jones, 2016). However, Jones (Jones, 2016) highlighted studies in which parents reported feeling powerless to prevent their adolescents from drinking, perceiving that their influence would be limited by external factors such as their children’s peers and cultural expectations (Roberts *et al*., 2010; Gilligan and Kypri, 2012). Jones (Jones, 2016) concluded that there was a need for further research that explores all predictors (attitudes, norms and perceived behavioural control) of the TPB. Jones also called for research that differentiates between adolescents of different ages given that alcohol initiation becomes more likely as adolescents become older (Jones, 2016).

### The social clock

Unlike most behaviours studied using the TPB, parental supply of alcohol and adolescent alcohol consumption more broadly have an additional characteristic that is likely to be associated with parents’ intentions: they are behaviours subject to the ‘social clock’, which becomes more acceptable as a child ages. Studies from Canada (Paglia and Room, 1998), the UK (Valentine *et al*., 2012), New Zealand (Kypri *et al*., 2007) and Australia (Jones *et al*., 2018) have found that the average age at which community members perceive that it is acceptable for children to drink a full drink of alcohol is at or slightly before the legal purchase age.

It is not yet known how views on an acceptable age of initiation are associated with Australian parents’ supply behaviours. When a behaviour is perceived to be acceptable, it may vary among parents based on factors such as their parenting style. For example, a permissive parenting style involves behaving in a non-punitive, accepting manner (Baumrind, 1968). Permissive parents may deem alcohol consumption acceptable at an earlier age than parents with styles more focused on controlling a child’s activities (authoritarian) or directing them through reasoning (authoritative). In this study, we examine Australian parents’ perceptions about the acceptable age of alcohol initiation and incorporate these perceptions within the broader TPB to examine associations with parental supply of alcohol.

### Research questions

(1) Does the age at which Australian parents consider it acceptable for their child to consume a full drink of alcohol differ by parenting style and adolescent age?
(2) How is parental supply of alcohol associated with attitudes toward adolescent alcohol use, perceived norms, perceived behavioural control and perceived acceptable age to drink a full drink of alcohol?

## METHODS

### Participants and procedure

This study utilized a cross-sectional 25-min online survey of 1197 parents or guardians of children aged 12–17 years old residing in Australia, and whose child lived with them at least some of the time. Participants were recruited in April 2022 from Pureprofile, an online social and market research panel consisting of people residing in Australia who have registered their interest in undertaking surveys. The panel provider invited panellists who had previously indicated that they had children aged 12–17 years to complete the survey, with eligibility confirmed at the commencement of the survey through screening questions regarding number and age of children who live with the respondent at least some of the time. Panellists were provided with information about the study and asked to indicate their consent to participate before completing the survey. Those who completed the survey and met the online panel provider’s data checks (which involved terminating respondents who move through a survey too quickly or provide patterned or unreliable responses) were reimbursed approximately AUD5 for their time. Demographic quotas were used to ensure that the sample included approximately equal numbers of mothers and fathers and that distribution of respondents across states and territories and from metropolitan and non-metropolitan areas was representative of the distribution of the Australian population residing in each jurisdiction/area (e.g. 25.2% of parents were from Victoria, and Victoria had 25.5% of Australia’s population in March 2022; Australian Bureau of Statistics, 2024). The study was approved by the Flinders University Human Ethics Low Risk Panel (app: 5020).

### Measures

Measures relevant to the current study aims are described here. The full questionnaire is available on the Open Science Framework (Bowden *et al*., 2022).

#### Demographics

Participants reported their own gender and age (in years), country of birth, household income (pre-tax), level of education, postcode and the gender and age range (12–15 years or 16–17 years) of their child. Based on the Australian Bureau of Statistics Greater Capital City Statistical Areas (Australian Bureau of Statistics, 2021), postcodes from capital cities were classified as ‘metropolitan’ and others as ‘non-metropolitan’.

#### Outcome variables

##### Acceptable age to drink a full drink of alcohol

Participants reported the age from which they thought it was acceptable to drink one (a) beer, (b) glass of wine, (c) pre-mixed spirit and (d) shot (or nip) of a spirit, in single years from age 0–25, ‘26 or older’, or ‘Never okay’ (adapted from Paglia and Room, 1998). The youngest age reported as acceptable across these items was taken as the acceptable age to drink a full drink of alcohol.

##### Parental supply of alcohol

Participants reported how often they gave their child a full drink of alcohol (a) at home with dinner, (b) at a family function directly supervised, (c) at home not directly supervised, and (d) to bring to a party not directly supervised, on a scale from 1 ‘Never’ to 6 ‘More than once a week’ (adapted from Gilligan *et al*., 2014; Jongenelis *et al*., 2018). Parents who responded ‘Never’ to all four items were classified as never providing a full drink of alcohol, others as providing a full drink. Participants also reported how often they gave their child alcohol to have as a sip/diluted drink under supervision, on the same scale. Parents who responded ‘Never’ to this item in addition the four items regarding provision of full drinks of alcohol were classified as never providing any alcohol, others as providing any alcohol (including sips).

#### TPB components

##### Attitudes toward adolescent alcohol use

Participants responded to 14 statements reflecting appraisals of adolescent alcohol use (shown in Supplementary Table S1) on a scale from 1 ‘Strongly disagree’ to 5 ‘Strongly agree’ (adapted from Crawford and Novak, 2006; Jongenelis *et al.*, 2018; Logi Kristjansson, 2019; Norris *et al.*, 2022). Based on a principal component analysis (detailed in Supplementary Materials), an ‘adverse effects’ score, reflecting appraisals of alcohol as harmful for adolescents (e.g. ‘Alcohol consumption during adolescence can affect teenage brain development’), was calculated as the mean of 10 items (Cronbach’s *α* = 0.88). A ‘social benefits’ score, reflecting appraisals of alcohol as socially beneficial for adolescents (e.g. ‘Drinking alcohol is important so that a teenager is not left out of their peer group’), was calculated as the mean of four items (Cronbach’s *α* = 0.84).

##### Perceived norms regarding parental supply of alcohol

Participants reported how often they thought that (a) their friends and (b) other parents gave alcohol to their adolescents of the same age to drink when unsupervised, with response options 1 ‘Less than me’, 2 ‘About the same as me’, or 3 ‘More than me’ (adapted from Gilligan *et al*., 2014). To derive an absolute measure of perceived norms, participants were coded as perceiving that friends/others did not supply alcohol for unsupervised use if they reported (a) ‘Never’ supplying alcohol to their adolescent to bring to a party not directly supervised and that friends/others supplied ‘Less than me’ or ‘About the same as me’, or (b) supplying alcohol in this context but that friends/others supplied ‘Less than me’. Participants were coded as perceiving that friends/others supplied alcohol for unsupervised use if they reported (a) ‘Never’ supplying alcohol in this context and that friends/others supplied ‘More than me’, or (b) supplying alcohol in this context and that friends/others supplied ‘About the same as me’ or ‘More than me’.

A perceived norm regarding parental supply in any setting (supervised or unsupervised) was also explored, as this would align more closely to the outcome variable. However, deriving this norm required the use of responses to the outcome variable, which may have overinflated associations between the variables. An analytic model using the broader norm fitted poorly, as shown by a significant Hosmer–Lemeshow test (*χ*^2^(8) = 44.53, *p* < 0.001, see Supplementary Table S2). Nonetheless, the direction and significance of association were consistent whether the unsupervised or broader norm were used.

##### Perceived behavioural control

Participants indicated the extent to which they agreed with the statement ‘In teaching my child about alcohol, I feel my influence as a parent will be overridden by the influence of Australian cultural expectations’ on a scale from 1 ‘Strongly disagree’ to 5 ‘Strongly agree’, collapsed to ‘Disagree’, ‘Neither agree nor disagree’, and ‘Agree’ for analyses (Roberts *et al*., 2010).

#### Modifiable behaviours and knowledge

##### Parenting style

The 30-item Parental Authority Questionnaire – Revised (Reitman *et al*., 2002) was used to measure participants’ parenting style. Participants were assigned a dominant parenting style of authoritative, authoritarian, or permissive (refer to Bartram *et al*., 2024 for details).

##### Parental alcohol consumption

The three-item Alcohol Use Disorders Identification Test—Consumption (AUDIT-C) (Bush *et al*., 1998) was used to measure risky alcohol consumption, with scores of 5 or greater categorized as ‘risky’ (Rumpf *et al*., 2002; Fischer *et al*., 2021).

##### Understanding of Australian Alcohol Guideline for people under 18 years of age

The relevant Australian Alcohol Guideline recommends that ‘To reduce the risk of injury and other harms to health, children and people under 18 years of age should not drink alcohol’ (National Health and Medical Research Council, 2020). Participants reported their understanding of the maximum number of alcoholic drinks per day a healthy adolescent (a) under 15 years of age and (b) aged 15–17 years can consume if they want to minimize the risks associated with alcohol consumption. Responding ‘0’ to both questions was categorized as correct understanding of the Guideline, while other responses (i.e. ≥1 to either question) were categorized as incorrect.

### Analyses

Analyses were performed using IBM SPSS Statistics 28 (IBM Corp, 2021). One hundred and fifteen respondents were excluded from analyses due to extreme responding (*Z*-score >3) on measures relating to acceptable age to drink a full drink of alcohol. The analytic sample size is *N* = 1082 except where noted (one participant had missing data for country of birth; otherwise, data were complete). Initial data exploration showed that acceptable age to drink a full drink of alcohol could not be treated as a continuous variable due to the narrow distribution of responses; responses were categorized as <16 years, 16–17 years, or ≥18 years (including ‘Never okay’).

Univariable analysis was performed using chi squared tests to identify potential predictors (at *p* < 0.05) for acceptable age to drink a full drink of alcohol and parental supply of a full drink of alcohol. To address research question 1, a multinomial logistic regression model was used to examine associations between acceptable age to drink a full drink of alcohol and parenting style, adolescent age, and other demographic, modifiable knowledge, and behavioural variables that showed a significant association with acceptable age in univariate analyses. To address research question 2, a binomial logistic regression model was used to examine associations between parental supply of a full drink of alcohol and predictor variables, including tailored TPB components (attitudes, norms, perceived behavioural control and acceptable age), demographic variables, and modifiable knowledge and behavioural variables previously shown to be associated with parental supply (Booth *et al*., 2023). A hierarchical analysis was conducted to examine the incremental variance explained (using Cox and Snell’s and Nagelkerke’s pseudo-*R*^2^ values) by the tailored TPB components. As a sensitivity analysis, the binomial logistic regression model was re-run using parental supply of any alcohol (including sips) as the dependent variable.

Analyses were pre-registered (Bowden *et al*., 2022), and deviated from the pre-registered plan as follows. In relation to research question 1, a multinomial logistic regression model was used instead of one-way ANCOVAs as it was necessary to treat acceptable age as a categorical variable. Research question 2 was not specified in the pre-registered plan; it reflects an evolution in conceptual thinking regarding the social clock and parental supply of alcohol. Analyses relating to this question should be considered exploratory.

## RESULTS

Sample characteristics are shown in Table 1. Respondents had a mean age of 45.8 years (SD 7.8), with just over half of respondents identifying as female (52.5%), reporting that their eldest adolescent was male (52.2%) and aged 12–15 years (59.7%). About three-quarters (76.1%) of respondents were born in Australia and 71.6% lived in a metropolitan area. Just under half (46.4%) of respondents reported having a university level of education and reported household income was relatively evenly distributed across income bands.

**Table 1:** Sample characteristics overall, by acceptable age and by provision of full drinks

Categorical variables				Acceptable age to drink a full drink of alcohol				Reported provision of full drinks		
			(*N* = 1082)	<16 (*N* = 124; 11.5%)	16–17 (*N* = 338; 31.2%)	≥18 (*N* = 620; 57.3%)	*p*	No (*N* = 837; 77.4%)	Yes (*N* = 245; 22.6%)	*p*
			%	%				%		
**Tailored TPB components**										
Perceive that parent friends supply alcohol for unsupervised use	No		70.7	**9.2**	**30.2**	**60.7**	**<0.001**	**86.8**	**13.2**	**<0.001**
	Yes		29.3	**17.0**	**33.8**	**49.2**		**54.6**	**45.4**	
Perceive that other parents supply alcohol for unsupervised use	No		55.0	**8.9**	**29.1**	**62.0**	**<0.001**	**88.4**	**11.6**	**<0.001**
	Yes		45.0	**14.6**	**33.9**	**51.5**		**63.9**	**36.1**	
Influence as a parent will be overridden by Australian cultural expectations	Disagree		26.9	10.3	30.2	59.5	0.624	79.4	20.6	0.241
	Neither agree nor disagree		32.4	12.8	29.3	57.8		78.9	21.1	
	Agree		40.7	11.1	33.4	55.5		74.8	25.2	
Acceptable age to drink a full drink of alcohol	≥18 years		57.3	—	—	—	—	**91.1**	**8.9**	**<0.001**
	16–17 years		31.2	—	—	—	—	**65.4**	**34.6**	
	<16 years		11.5	—	—	—	—	**41.1**	**58.9**	
**Demographics**										
Adolescent age	12–15 years		59.7	12.1	29.6	58.4	0.321	**85.6**	**14.4**	**<0.001**
	16–17 years		40.3	10.6	33.7	55.7		**65.1**	**34.9**	
Adolescent gender	Male		52.2	13.5	29.6	57.0	0.075	75.8	24.2	0.187
	Female		47.8	9.3	33.1	57.6		79.1	20.9	
Parent gender	Male		47.5	12.8	28.4	58.8	0.105	**74.7**	**25.3**	**0.048**
	Female		52.5	10.2	33.8	56.0		**79.8**	**20.2**	
Country of birth^†^	Australia		76.1	11.3	32.8	55.9	0.115	**75.8**	**24.2**	**0.034**
	Elsewhere		23.9	12.0	26.0	62.0		**82.2**	**17.8**	
Income	<$60 000		22.6	**13.9**	**24.9**	**61.2**	**0.044**	82.0	18.0	0.065
	$60 000–<$100 000		27.6	**11.7**	**28.8**	**59.5**		77.3	22.7	
	$100 000–<$150 000		28.4	**11.7**	**33.6**	**54.7**		72.6	27.4	
	≥$150 000		21.3	**8.2**	**38.1**	**53.7**		78.8	21.2	
Level of education	No university		53.6	13.1	32.4	54.5	0.073	77.6	22.4	0.846
	University		46.4	9.6	29.9	60.6		77.1	22.9	
Region	Metropolitan		71.6	**10.2**	**29.5**	**60.3**	**0.005**	78.5	21.5	0.172
	Non-metropolitan		28.4	**14.7**	**35.5**	**49.8**		74.6	25.4	
**Modifiable behaviours and knowledge**										
Understanding of guideline for under 18s	Incorrect		22.8	**25.9**	**34.8**	**39.3**	**<0.001**	**62.3**	**37.7**	**<0.001**
	Correct		77.2	**7.2**	**30.2**	**62.6**		**81.8**	**18.2**	
Parental risky drinking	No		65.7	11.1	29.3	59.6	0.091	**81.4**	**18.6**	**<0.001**
	Yes		34.3	12.1	35.0	52.8		**69.5**	**30.5**	
Parenting style	Authoritative		72.2	10.9	32.5	56.6	0.503	**80.3**	**19.7**	**<0.001**
	Authoritarian		16.6	11.7	28.9	59.4		**77.2**	**22.8**	
	Permissive		11.2	14.9	26.4	58.7		**58.7**	**41.3**	

Continuous variables	Mean	SD	Range	Mean				Mean		
**Tailored TPB components**										
Adverse effects	4.13	0.63	2–5	**3.76**	**4.04**	**4.26**	**<0.001**	**4.23**	**3.80**	**<0.001**
Social benefits	2.21	0.89	1–5	**2.64**	**2.35**	**2.04**	**<0.001**	**2.07**	**2.67**	**<0.001**
**Demographics**										
Parent age	45.76	7.82	26–81	44.71	45.48	46.12	0.137	45.75	45.76	0.993

Note: TPB = Theory of Planned Behaviour. *p* denotes the *p*-value for a two-sided Pearson Chi-Square test of independence for categorical variables and a *t*-test or one-way ANOVA for continuous variables. *N* = 1082 except for country of birth (*N* = 1081 due to missing response). Bold values indicate *p* < 0.05.

Almost one-quarter (22.6%) of parents in this sample had previously supplied a full drink of alcohol to their adolescent, with 19.1% reporting supplying drinks to drink while supervised at a family function/special occasion, 15.2% to drink at home with dinner, 10.8% to take to parties or special occasions unsupervised and 10.0% to drink at home unsupervised. Just over half (57.3%) of respondents nominated an acceptable age to drink a full drink of alcohol of 18 years or older (the legal purchase age in Australia), while 31.2% nominated 16 or 17 years and 11.5% nominated an age before 16 years. Nearly one-third (29.3%) of respondents perceived that their parent friends supply alcohol for unsupervised use, while nearly half (45.0%) perceived that other parents supply alcohol for unsupervised use—substantially more than the 10.8% of respondents who reported supply in this context. Two-fifths (40.7%) agreed that their influence as a parent will be overridden by Australian cultural expectations. The mean ‘adverse effects’ score was 4.13/5 (SD 0.63), indicating that on average, respondents appraised alcohol as harmful for adolescents’ health. The mean ‘social benefits’ score was 2.21/5 (SD 0.89), indicating that on average, respondents did not appraise alcohol as socially beneficial for adolescents.

Table 2 presents a multinomial logistic regression analysis examining associations between perceptions about the acceptable age to drink a full drink of alcohol and adolescent age, parenting style, and other demographic, knowledge, and behavioural variables significantly associated with acceptable age to drink in univariable analyses, namely income, region and understanding of the Australian Alcohol Guideline for people under 18 years of age (Table 1). Adolescent age and parenting style were not associated with perceptions about the acceptable age to drink a full drink of alcohol in either univariable or multivariable analyses. In the multinomial regression model, an incorrect understanding of the Guideline was significantly associated with perceptions that the acceptable age to drink alcohol was under 16 years (compared to ≥18 years, relative risk ratio [RRR] = 6.22, 95% CI = 4.06, 9.52) or 16 to 17 years (compared to ≥18 years, RRR = 2.00, 95% CI = 1.34, 2.80). Significant associations were also found with income, where respondents with household incomes above $100 000 (compared to <$60 000) were more likely to perceive that the acceptable age to drink a full drink of alcohol was 16 to 17 years (compared to ≥18 years, $100 000–<$150 000: RRR = 1.59, 95% CI = 1.07, 2.36; ≥$150 000: RRR = 1.90, 95% CI = 1.25–2.87). Being from a non-metropolitan rather than a metropolitan area was also significantly associated with perceptions that the acceptable age to drink alcohol was under 16 years (compared to ≥18 years, RRR = 2.04, 95% CI = 1.31, 3.17) or 16 to 17 years (compared to ≥18 years, RRR = 1.66, 95% CI = 1.23–2.25). As a sensitivity analysis, the model was re-run including children aged over 18 years; child age was still not associated with perceptions about the acceptable age to drink a full drink of alcohol (see Supplementary Table S3).

**Table 2:** Multinomial logistic regression analysis: acceptable age to drink a full drink of alcohol (reference category: 18 years or older)

Independent variables	<16 years	16–17 years	*p*
	RRR (95% CI)	RRR (95% CI)	
Adolescent age			0.365
12–15 years	Ref		
16–17 years	0.98 (0.64, 1.48)	1.20 (0.92–1.58)	
Parenting style			0.560
Authoritarian	Ref		
Authoritative	1.09 (0.63–1.89)	1.24 (0.85–1.80)	
Permissive	1.04 (0.50–2.17)	0.89 (0.51–1.54)	
Understanding of Australian alcohol guideline for <18 years			**<0.001**
Yes	Ref		
No	**6.22 (4.06–9.52)**	**2.00 (1.43–2.80)**	
Income			**0.029**
<$60 000	Ref		
$60 000–≤$100 000	0.91 (0.53–1.57)	1.23 (0.82–1.83)	
$100 000–≤$150 000	1.01 (0.58–1.76)	**1.59 (1.07–2.36)**	
≥$150 000	0.76 (0.40–1.45)	**1.90 (1.25–2.87)**	
Region			**<0.001**
Metropolitan	Ref		
Non-metropolitan	**2.04 (1.31–3.17)**	**1.66 (1.23–2.25)**	

Note: RRR = relative risk ratio; Ref = reference category. *N* = 1082. Bold values indicate *p* < 0.05.

Table 3 presents the results of a logistic regression analysis examining associations between provision of full drinks to adolescents and tailored TPB components, as well as demographic and modifiable behaviour and knowledge variables that showed a significant association with provision of full drinks in univariable analyses, namely adolescent age, parent gender, country of birth, risky parental alcohol consumption, parenting style and understanding of the Australian Alcohol Guideline for people under 18 years of age (Table 1). In the full model, variables significantly associated with *increased* odds of providing full drinks were appraisals of alcohol as socially beneficial for adolescents (adjusted odds ratio [AOR] = 1.31, 95% CI = 1.02, 1.69); perceptions that parent friends (AOR = 2.91, 95% CI = 1.80, 4.70) and other parents (AOR = 2.23, 95% CI = 1.37, 3.62) provide alcohol to adolescents for unsupervised use; perceptions that the acceptable age to drink a full drink of alcohol was under 16 years (AOR = 14.75, 95% CI = 8.23, 26.42) or 16 to 17 years (AOR = 5.68, 95% CI = 3.69, 8.73) compared to 18 years or older; having an adolescent aged 16 to 17 years (AOR = 3.33, 95% CI = 2.26, 4.90) compared to 12 to 15 years; risky parental alcohol consumption (AOR = 1.70, 95% CI = 1.15, 2.52); and a permissive (AOR = 2.45, 95% CI = 1.33, 4.49), compared to authoritative, but not an authoritarian parenting style. Appraisals of alcohol as harmful for adolescents were significantly associated with *reduced* odds of provision of full drinks (AOR = 0.49, 95% CI = 0.36, 0.68). Perceived behavioural control was not significantly associated with provision of full drinks to adolescents in this multivariable model; nor were country of birth, parent gender or understanding of the Australian Alcohol Guideline for people under 18 years of age. A hierarchical logistic regression analysis (Supplementary Table S4) showed that tailored TPB components explained significant additional variance in the provision of full drinks beyond that explained by the demographic and modifiable behaviour and knowledge variables, increasing Cox and Snell *R*^2^ from 0.12 to 0.32 and Nagelkerke *R*^2^ from 0.19 to 0.49 (Step test: *χ*^2^(8) = 279.82, *p* < 0.001).

**Table 3:** Logistic regression analyses: provision of full drinks to adolescents (vs. reference category = no provision of full drinks)

Independent variables		AOR	95% CI	*p*
Tailored TPB components				
*Attitudes*				
Adverse effects (continuous variable)		**0.49**	**0.36, 0.68**	**<0.001**
Social benefits (continuous variable)		**1.31**	**1.02, 1.69**	**<0.001**
*Perceived norms*				
Perceive that parent friends supply alcohol for unsupervised use	No	Ref		**<0.001**
	Yes	**2.91**	**1.80, 4.70**	
Perceive that other parents supply alcohol for unsupervised use	No	Ref		**0.001**
	Yes	**2.23**	**1.37, 3.62**	
*Perceived behavioural control*				
Influence as a parent will be overridden by Australian cultural expectations	Disagree	Ref		0.351
	Neither agree nor disagree	0.70	0.43, 1.15	
	Agree	0.87	0.54, 1.40	
*Social clock*				
Acceptable age to consume a full drink of alcohol	≥18 years	Ref		**<0.001**
	16–17 years	**5.68**	**3.69, 8.73**	
	<16 years	**14.75**	**8.23, 26.42**	
Demographics				
Adolescent age	12–15 years	Ref		**<0.001**
	16–17 years	**3.33**	**2.26, 4.90**	
Country of birth	Australia	Ref		0.356
	Elsewhere	0.81	0.51, 1.27	
Parent gender	Male	Ref		0.847
	Female	0.96	0.65, 1.43	
Modifiable behaviours and knowledge				
Parent risky alcohol consumption	No	Ref		**0.008**
	Yes	**1.70**	**1.15, 2.52**	
Understanding of Australian alcohol guideline for <18 years	Correct	Ref		0.856
	Incorrect	1.04	0.67, 1.62	
Parenting style	Authoritative	Ref		**0.015**
	Authoritarian	1.31	0.79, 2.18	
	Permissive	**2.45**	**1.33, 4.49**	

Note: AOR = adjusted odds ratio; TPB = Theory of Planned Behaviour; Ref = reference category. *N* = 1081 due to missing response on country of birth. Bold values indicate *p* < 0.05.

### Sensitivity analysis: sips versus full drinks

Results of the logistic regression model with provision of any alcohol (including sips) as the dependent variable are shown in Supplementary Table S5. These follow the same pattern as supply of full drinks except that the associations with appraisals of alcohol as socially beneficial (*p = *0.056) and parenting style (*p = *0.916) were non-significant.

## DISCUSSION

This is the first study to examine how parents’ attitudes and perceptions were associated with supply of alcohol to adolescents using a tailored TPB incorporating the concept of the social clock. Consistent with earlier studies (Ward and Snow, 2011a; Jones, 2016), parents’ attitudes were associated with parental supply of alcohol, with parents who appraised alcohol as having adverse effects for adolescents being less likely to supply alcohol, while those who appraised alcohol as having social benefits for adolescents being more likely to supply alcohol. Perceived norms around parental supply were also associated with parents’ own supply behaviours. There may be considerable misperceptions about the prevalence of parental supply among parents: in this sample, 11% of parents indicated that they provide alcohol for unsupervised use, despite 45% perceiving that other parents supplied alcohol in this context. This is consistent with Jones and Francis’ finding that parents perceived community views on parental supply of alcohol to be more liberal than their own (Jones and Francis, 2015). The indicator for perceived behavioural control was not found to be associated with parental supply in this study. Although consistent with Jones’ conceptualization of perceived behavioural control, this indicator referred to control over adolescents’ alcohol consumption, rather than adolescents’ access to alcohol (Jones, 2016). Perceptions about parents’ influence over their adolescents’ access to alcohol specifically, may be more strongly associated with parental supply—a potential direction for future research.

This study makes a novel contribution to the literature by examining the concept of the social clock in relation to parental supply of alcohol. As in international studies (Kypri *et al*., 2007; Valentine *et al*., 2012; Jones *et al*., 2018), the majority of parents nominated the age of legal purchase, 18 years of age in Australia, as the age at which it is acceptable to drink a full drink of alcohol. However, a substantial minority of parents nominated an age earlier than the age of legal purchase, with these parents being more likely to supply alcohol to their adolescents. In contrast to expectations, perceptions about acceptable age were not associated with parenting style—instead, these two variables were independently associated with parental supply. This suggests that permissive and authoritative parents may differ less on their perceptions about acceptable age to drink alcohol than on the extent to which they allow other considerations consistent with their parenting style, such as a desire to maintain a friendly relationship with their child for permissive parents, to guide their behaviours.

Perceptions about acceptable age were associated with household income and geographic location. The finding that higher incomes were associated with an increased likelihood of perceiving that it is acceptable to drink alcohol at 16 or 17 years of age is consistent with previous findings that people from higher socioeconomic status backgrounds have more tolerant attitudes toward young people’s alcohol consumption (Paglia and Room, 1998). However, it appears there may be a limit to this tolerance, with income not associated with perceptions that it is acceptable to drink alcohol before 16 years of age. This age may reflect the point at which concerns about increased risk to a young person’s health outweigh higher-income parents’ otherwise liberal attitudes toward young people’s behaviour. Future research might examine whether similar limits are observable for other contested behaviours (e.g. vaping, using marijuana). Interestingly, being from a non-metropolitan location was associated with perceptions that it is acceptable to drink alcohol before 18 years of age, but not parental supply. This may indicate that parental perceptions of age-appropriate alcohol consumption, more so than direct supply of alcohol by parents, may be shaping higher rates of adolescent alcohol use in non-metropolitan Australian communities (cf. Chan *et al*., 2016).

Regarding demographics and modifiable risk factors beyond TPB components, similar to previous studies, this study found that parental supply was associated with permissive parenting (Booth *et al*., 2023), parental alcohol use (Ward and Snow, 2011b; Booth *et al*., 2023), and older adolescent age (Ward and Snow, 2011b; Jongenelis *et al*., 2018). In contrast to Booth *et al.*, we found no association with parent’s age, while understanding of the Australian Alcohol Guidelines for people aged under 18 showed a bivariate association but was not significant in the multivariate model (Booth *et al*., 2023). Understanding of the Guideline was, however, associated with perceptions about acceptable age, which in turn was associated with parental supply. TPB components explained substantial additional variance in parental supply beyond demographics and modifiable behaviours and attitudes, suggesting that these components may be more proximal determinants of parental supply.

### Limitations

This study included a large, diverse sample of parents of adolescents living in Australia, with quotas used to strengthen the sample’s representativeness. However, as the sample was recruited from an online social and market research panel, findings may not be generalizable. Responses may have been affected by social desirability, although the survey’s anonymity was emphasized, and previous qualitative research has demonstrated that parents will openly discuss their supply of alcohol (Norris *et al.*, 2022). The measure of perceived norms of parental supply for unsupervised use did not directly align with the outcome variable of parental supply in any context, which may have attenuated the strength of association between perceived norms and parental supply behaviour. As a cross-sectional survey, the direction of association between variables is not clear, and it is possible that parents’ supply behaviours may have influenced their attitudes and perceptions—which is consistent with the TPB (Ajzen, 1991). There is a need for longitudinal research to explore whether attitudes, norms and perceptions of the acceptable age of alcohol consumption change over time or predict later parental supply behaviour.

### Implications for practice

Parental supply of alcohol to adolescents is common in Australia and in many countries across the world (van der Kruk *et al*., 2023), prompting a need to develop health promotion interventions to reduce parental supply of alcohol. This study’s findings suggest that interventions could target parents’ attitudes by increasing awareness of the adverse effects of adolescent alcohol consumption and challenge perceptions that there are benefits of alcohol use in adolescence, as well as highlighting that parental supply of alcohol is not a normative practice (van der Kruk *et al*., 2023). Interventions could also aim to increase parents’ awareness of the Australian Alcohol Guideline for people under 18 years of age. Although an understanding of the Guideline was not directly associated with parental supply in the full model, increasing awareness may be a potential way to influence parents’ perceptions of the acceptable age for alcohol consumption. Further research using experimental designs will be needed to test whether interventions targeting these tailored TPB concepts can effectively discourage parents from supplying alcohol to their adolescents, thereby reducing adolescent harms from alcohol consumption.

## Supplementary Material

daae173_suppl_Supplementary_Tables_1-5

## Data Availability

The data underlying this article will be shared on reasonable request to the corresponding author.
