# Supplementary material for: Australian parents’ attitudes, perceptions and supply of alcohol to adolescents: a national cross-sectional survey
Source: Health Promot Int. 2024 Dec 9;39(6):daae173. doi: 10.1093/heapro/daae173 (PMC11631088; doi:10.1093/heapro/daae173)
Supplement: daae173_suppl_Supplementary_Tables_1-5 [file daae173_suppl_supplementary_tables_1-5.docx]

**Supplementary Material**

**Attitudes toward adolescent alcohol use: Principal component analysis**

A principal component analysis with orthogonal (varimax) rotation was conducted to explore whether 14 statements used in the full study survey and capturing attitudes toward adolescent alcohol use could be reduced into fewer components. Both Kaiser’s criterion (eigenvalues >1) and a visual inspection of the scree plot supported retaining two components in the final analysis. Supplementary Table 1 shows the factor loadings on these components after rotation. The items that cluster on the same components suggest that component 1 represents *beliefs that alcohol is harmful for adolescents’ health*, while component 2 represents *beliefs that alcohol has social benefits for adolescents*. Based on this analysis, a ‘adverse effects’ score was computed as the mean of items 1 to 10 (Cronbach’s α = .88) and a ‘social benefits’ score was computed as the mean of items 11 to 14 (Cronbach’s α =.84). Both scores were treated as continuous variables in further analyses.

**Supplementary Table 1.** Summary of principal component analysis results for attitudes toward adolescent alcohol use items (N=1,082).

|  |  |  | Rotated Factor Loadings | |
| --- | --- | --- | --- | --- |
| Item | Mean | SD | Adverse Effects | Social Benefits |
| 1. Alcohol can increase a young person’s risk of developing harmful drinking behaviour as an adult | 4.18 | 0.89 | **.74** | -.15 |
| 2. Alcohol can affect a young person’s mood and mental health | 4.33 | 0.78 | **.73** | -.30 |
| 3. Alcohol can affect a young person’s memory, ability to learn, and problem-solving skills | 4.25 | 0.81 | **.73** | -.29 |
| 4. Alcohol consumption during adolescence can affect teenage brain development | 4.29 | 0.81 | **.73** | -.26 |
| 5. Alcohol can increase a young person’s risk of antisocial behaviour | 4.16 | 0.90 | **.72** | -.10 |
| 6. Alcohol can make adolescents more likely to engage in risky sexual behaviour | 4.27 | 0.81 | **.71** | -.14 |
| 7. Alcohol can increase a young person’s risk of accident and injury | 4.39 | 0.78 | **.69** | -.25 |
| 8. The younger a person starts drinking, the more likely they are to develop risky drinking patterns | 4.08 | 0.98 | **.68** | -.13 |
| 9. Exposing young people to alcohol use encourages them to drink more alcohol from an earlier age | 3.61 | 1.12 | **.62** | .06 |
| 10. Two to three drinks on an occasion harms the health of a teenager | 3.77 | 1.02 | **.57** | -.12 |
| 11. Drinking alcohol is important so that a teenager is not left out of their peer group | 2.10 | 1.07 | -.17 | **.83** |
| 12. Alcohol will help my child socialise better | 2.05 | 1.05 | -.18 | **.80** |
| 13. Drinking makes for great stories and offers the best memories of adolescent life | 2.25 | 1.09 | -.14 | **.79** |
| 14. Drinking alcohol is an important rite of passage for a young person | 2.42 | 1.13 | -.16 | **.78** |
| Eigenvalues |  |  | 4.90 | 2.95 |
| % of variance |  |  | 34.99 | 21.06 |
| Cronbach’s α |  |  | .88 | .84 |

Note: Participant responses were from 1 ‘Strongly disagree’ to 5 ‘Strongly agree’. Primary factor loadings appear in bold. KMO=.93. Bartlett’s test of sphericity χ^2^(91)=6535.19, *p* < .001.

**Supplementary Table 2.** Logistic regression analyses: Provision of full drinks to adolescents (using perceived norm of supply in any setting) (N=1081)

| **Independent variables** | | **AOR** | **95% CI** | ***p*** |
| --- | --- | --- | --- | --- |
| **Tailored TPB components** | |  |  |  |
| ***Attitudes*** | |  |  |  |
| Adverse effects (continuous variable) | | **0.46** | **0.31, 0.67** | **<.001** |
| Social benefits (continuous variable) | | **1.69** | **1.24, 2.31** | **<.001** |
| ***Perceived norms*** | |  |  |  |
| Perceive that parent friends supply alcohol | No | Ref |  |  |
|  | Yes | **15.78** | **8.39, 29.69** | **<.001** |
| Perceive that other parents supply alcohol | No | Ref |  |  |
|  | Yes | **5.11** | **2.53, 10.33** | **<.001** |
| ***Perceived behavioural control*** | |  |  |  |
| Influence as a parent will be overridden by Australian cultural expectations | Disagree | Ref |  |  |
|  | Neither agree nor disagree | 0.83 | 0.46, 1.49 | .521 |
|  | Agree | 0.92 | 0.52, 1.62 | .771 |
| ***Social clock*** | |  |  |  |
| Acceptable age to consume a full drink of alcohol | ≥18 years | Ref |  |  |
|  | 16-17 years | **6.23** | **3.76, 10.32** | **<.001** |
|  | <16 years | **16.71** | **8.20, 34.03** | **<.001** |
| **Demographics** | |  |  |  |
| Adolescent age | 12-15 years | Ref |  |  |
|  | 16-17 years | **2.78** | **1.75, 4.42** | **<.001** |
| Country of birth | Australia | Ref |  |  |
|  | Elsewhere | 0.79 | 0.46, 1.34 | .381 |
| Parent gender | Male | Ref |  |  |
|  | Female | 0.96 | 0.59, 1.54 | .854 |
| **Modifiable behaviours and knowledge** | |  |  |  |
| Risky alcohol consumption | No | Ref |  |  |
|  | Yes | 1.49 | 0.94, 2.38 | .089 |
| Understanding of Australian alcohol guideline for <18 years | Correct | Ref |  |  |
|  | Incorrect | 1.19 | 0.70, 2.04 | .521 |
| Parenting style | Authoritative | Ref |  |  |
|  | Authoritarian | 1.56 | 0.84, 2.88 | .157 |
|  | Permissive | 3.84 | 1.84, 7.99 | .384 |

Note. AOR = Adjusted Odds Ratio, TPB = Theory of Planned Behaviour. N=1081 due to missing response on country of birth. Model statistics: χ^2^(15) = 626.26, *p* < .001; Cox & Snell *R*^2^ = 0.44; Nagelkerke R^2^ = 0.67; Hosmer-Lemeshow goodness of fit test, *p* < .001. Bold values indicate *p* < .05.

**Supplementary Table 3.** Multinomial logistic regression analysis: Acceptable age to drink a full drink of alcohol (reference category: 18 years or older)

| **Independent variables** | **<16 years** | **16-17 years** |  |
| --- | --- | --- | --- |
|  | **RRR (95% CI)** | **RRR (95% CI)** | ***p*** |
| Child age |  |  | .566 |
| 12-15 years | Ref |  |  |
| 16-17 years | 0.95 (0.54, 1.65) | 1.04 (0.73, 1.49) |  |
| 18 years or older | 1.27 (0.81, 1.99) | 1.25 (0.92, 1.70) |  |
| Parenting style |  |  | .527 |
| Authoritarian | Ref |  |  |
| Authoritative | 1.10 (0.64, 1.91) | 1.26 (0.87, 1.83) |  |
| Permissive | 1.05 (0.50, 2.19) | 0.90 (0.52, 1.55) |  |
| Understanding of Australian alcohol guideline for <18 years |  |  | **<.001** |
| Yes | Ref |  |  |
| No | **6.18 (4.03, 9.46)** | **1.99 (1.42, 2.78)** |  |
| Income |  |  | **.026** |
| < $60,000 | Ref |  |  |
| $60,000-≤$100,000 | 0.92 (0.53, 1.59) | 1.24 (0.83, 1.85) |  |
| $100,000-≤$150,000 | 1.02 (0.59, 1.77) | **1.61 (1.09, 2.39)** |  |
| ≥$150,000 | 0.76 (0.40, 1.45) | **1.91 (1.26, 2.89)** |  |
| Region |  |  | **<.001** |
| Metropolitan | Ref |  |  |
| Non-metropolitan | **2.03 (1.30, 3.16)** | **1.65 (1.22, 2.24)** |  |

Note: RRR = Relative Risk Ratio, Ref = reference category. N=1082. Bold values indicate *p*<.05.

**Supplementary Table 4.** Logistic regression analyses: Provision of full drinks to adolescents (N=1081)

| Independent variables | | Step 1: Demographics | | | Step 2: Modifiable behaviours and knowledge | | | Step 3: Extended TPB components | | |
| --- | --- | --- | --- | --- | --- | --- | --- | --- | --- | --- |
|  |  | AOR | 95% CI | *p* | AOR | 95% CI | *p* | AOR | 95% CI | *p* |
| **Demographics** | |  |  |  |  |  |  |  |  |  |
| Adolescent age | 12-15 years | Ref |  | <.001 | Ref |  | <.001 | Ref |  | <.001 |
|  | 16-17 years | **3.11** | **2.31, 4.19** |  | **3.58** | **2.61, 4.90** |  | **3.33** | **2.26, 4.90** |  |
| Country of birth | Australia | Ref |  | .039 | Ref |  | .042 | Ref |  | .356 |
|  | Elsewhere | **0.68** | **0.47, 0.98** |  | **0.67** | **0.46, 0.99** |  | 0.81 | 0.51, 1.27 |  |
| Parent gender | Male | Ref |  | .128 | Ref |  | .774 | Ref |  | .847 |
|  | Female | 0.79 | 0.59, 1.07 |  | 1.05 | 0.76, 1.44 |  | 0.96 | 0.65, 1.43 |  |
|  | |  |  |  |  |  |  |  |  |  |
| **Modifiable behaviours and knowledge** | |  |  |  |  |  |  |  |  |  |
| Risky alcohol consumption | No |  |  |  | Ref |  | .003 | Ref |  | .008 |
|  | Yes |  |  |  | **1.64** | **1.19, 2.27** |  | **1.70** | **1.15, 2.52** |  |
| Understanding of Australian alcohol guideline for <18 years | Yes |  |  |  | Ref |  | <.001 | Ref |  | .856 |
|  | No |  |  |  | **2.75** | **1.95, 3.87** |  | 1.04 | 0.67, 1.62 |  |
| Parenting style | Authoritative |  |  |  | Ref |  | <.001 | Ref |  | .015 |
|  | Authoritarian |  |  |  | 1.12 | 0.73, 1.70 |  | 1.31 | 0.79, 2.18 |  |
|  | Permissive |  |  |  | **2.71** | **1.74, 4.20** |  | **2.45** | **1.33, 4.49** |  |
|  | |  |  |  |  |  |  |  |  |  |
| **Extended TPB components** | |  |  |  |  |  |  |  |  |  |
| ***Attitudes*** | |  |  |  |  |  |  |  |  |  |
| Adverse effects (continuous variable) | |  |  |  |  |  |  | **0.49** | **0.36, 0.68** | <.001 |
| Social benefits (continuous variable) | |  |  |  |  |  |  | **1.31** | **1.02, 1.69** | <.001 |

| Independent variables | | Step 1: Demographics | | | Step 2: Modifiable behaviours and knowledge | | | Step 3: Extended TPB components | | |
| --- | --- | --- | --- | --- | --- | --- | --- | --- | --- | --- |
|  |  | AOR | 95% CI | *p* | AOR | 95% CI | *p* | AOR | 95% CI | *p* |
| ***Perceived norms*** | |  |  |  |  |  |  |  |  |  |
| Perceive that parent friends supply alcohol for unsupervised use | No |  |  |  |  |  |  | Ref |  | **<.001** |
|  | Yes |  |  |  |  |  |  | **2.91** | **1.80, 4.70** |  |
| Perceive that other parents supply alcohol for unsupervised use | No |  |  |  |  |  |  | Ref |  | **.001** |
|  | Yes |  |  |  |  |  |  | **2.23** | **1.37, 3.62** |  |
| ***Perceived behavioural control*** | |  |  |  |  |  |  |  |  |  |
| Influence as a parent will be overridden by Australian cultural expectations | Disagree |  |  |  |  |  |  | Ref |  | .351 |
|  | Neither agree nor disagree |  |  |  |  |  |  | 0.70 | 0.43, 1.15 |  |
|  | Agree |  |  |  |  |  |  | 0.87 | 0.54, 1.40 |  |
| ***Social clock*** | |  |  |  |  |  |  |  |  |  |
| Acceptable age to consume a full drink of alcohol | 18 years or older |  |  |  |  |  |  | Ref |  | **<.001** |
|  | 16 or 17 years |  |  |  |  |  |  | **5.68** | **3.69, 8.73** |  |
|  | Before 16 years |  |  |  |  |  |  | **14.75** | **8.23, 26.42** |  |
|  |  |  |  |  |  |  |  |  |  |  |
| **Overall model statistics** | |  |  |  |  |  |  |  |  |  |
|  | | **χ^2^** | ***df*** | ***p*** | **χ^2^** | ***df*** | ***p*** | **χ^2^** | ***df*** | ***p*** |
| Model test | | 67.82 | 3 | <.001 | 140.12 | 7 | <.001 | 419.95 | 15 | <.001 |
| Step test | | 67.82 | 3 | <.001 | 72.3 | 4 | <.001 | 279.82 | 8 | <.001 |
| Hosmer-Lemeshow goodness of fit test | | 1.13 | 5 | .952 | 4.98 | 7 | .662 | 12.52 | 8 | .130 |
|  | | ***R*^2^** |  |  | ***R*^2^** |  |  | ***R*^2^** |  |  |
| Cox & Snell *R*^2^ | | .06 |  |  | .12 |  |  | .32 |  |  |
| Nagelkerke *R*^2^ | | .09 |  |  | .19 |  |  | .49 |  |  |
|  | |  |  |  |  |  |  |  |  |  |

Note. AOR = Adjusted Odds Ratio, TPB = Theory of Planned Behaviour, Ref = reference category. Bold values indicate *p* < .05.

**Supplementary Table 5**. Logistic regression analyses: Provision of any alcohol, including sips, to adolescents

| **Independent variables** | | **AOR** | **95% CI** | ***p*** |
| --- | --- | --- | --- | --- |
| **Tailored TPB components** | |  |  |  |
| ***Attitudes*** | |  |  |  |
| Adverse effects (continuous variable) | | **0.64** | **0.49, 0.84** | **.001** |
| Social benefits (continuous variable) | | 1.22 | 0.99, 1.50 | .056 |
| ***Perceived norms*** | |  |  |  |
| Perceive that parent friends supply alcohol for unsupervised use | No | Ref |  | **<.001** |
|  | Yes | **1.98** | **1.32, 2.95** |  |
| Perceive that other parents supply alcohol for unsupervised use | No | Ref |  | **.003** |
|  | Yes | **1.78** | **1.23, 1.78** |  |
| ***Perceived behavioural control*** | |  |  |  |
| Influence as a parent will be overridden by Australian cultural expectations | Disagree | Ref |  | .358 |
|  | Neither agree nor disagree | 0.75 | 0.51, 1.12 |  |
|  | Agree | 0.89 | 0.61, 1.30 |  |
| ***Social clock*** | |  |  |  |
| Acceptable age to consume a full drink of alcohol | ≥18 years | Ref |  | **<.001** |
|  | 16-17 years | **4.30** | **3.11, 5.96** |  |
|  | <16 years | **7.22** | **4.41, 11.85** |  |
| **Demographics** | |  |  |  |
| Adolescent age | 12-15 years | Ref |  | **<.001** |
|  | 16-17 years | **2.19** | **1.61, 2.98** |  |
| Country of birth | Australia | Ref |  | .053 |
|  | Elsewhere | 0.70 | 0.49, 1.00 |  |
| Parent gender | Male | Ref |  | .554 |
|  | Female | 0.91 | 0.66, 1.25 |  |
| **Modifiable behaviours and knowledge** | |  |  |  |
| Risky alcohol consumption | No | Ref |  | **.027** |
|  | Yes | **1.43** | **1.04, 1.97** |  |
| Understanding of Australian alcohol guideline for <18 years | Correct | Ref |  | .079 |
|  | Incorrect | 1.39 | 0.96, 2.01 |  |
| Parenting style | Authoritative | Ref |  | .916 |
|  | Authoritarian | 0.92 | 0.61, 1.38 |  |
|  | Permissive | 0.96 | 0.57, 1.62 |  |

Note. AOR = Adjusted Odds Ratio, TPB = Theory of Planned Behaviour. N=1081 due to missing response on country of birth. Model statistics: χ^2^(15) = 335.27, *p* < .001; Cox & Snell *R*^2^ = 0.27; Nagelkerke *R*^2^ = 0.37; Hosmer-Lemeshow goodness of fit test, *p* = .04. Bold values indicate *p* < .05.
